# Implications of global warming for the climate of African rainforests

**DOI:** 10.1098/rstb.2012.0298

**Published:** 2013-09-05

**Authors:** Rachel James, Richard Washington, David P. Rowell

**Affiliations:** 1 Climate Research Lab, Oxford University Centre for the Environment, South Parks Road, Oxford OX1 3QY, UK; 2 Met Office Hadley Centre, FitzRoy Road, Exeter EX1 3PB, UK

**Keywords:** climate change, global warming, Central Africa, precipitation, projections, Indian Ocean

## Abstract

African rainforests are likely to be vulnerable to changes in temperature and precipitation, yet there has been relatively little research to suggest how the regional climate might respond to global warming. This study presents projections of temperature and precipitation indices of relevance to African rainforests, using global climate model experiments to identify local change as a function of global temperature increase. A multi-model ensemble and two perturbed physics ensembles are used, one with over 100 members. In the east of the Congo Basin, most models (92%) show a wet signal, whereas in west equatorial Africa, the majority (73%) project an increase in dry season water deficits. This drying is amplified as global temperature increases, and in over half of coupled models by greater than 3% per °C of global warming. Analysis of atmospheric dynamics in a subset of models suggests that this could be partly because of a rearrangement of zonal circulation, with enhanced convection in the Indian Ocean and anomalous subsidence over west equatorial Africa, the Atlantic Ocean and, in some seasons, the Amazon Basin. Further research to assess the plausibility of this and other mechanisms is important, given the potential implications of drying in these rainforest regions.

## 1. Introduction

Changes in local temperature and precipitation have the potential to affect African rainforests and have led to large ecological shifts on millennial timescales [1]. Projecting the impact of anthropogenic interference on the climate of West and Central Africa is therefore important: to provide information for forest conservation and adaptation, and also for mitigation, allowing assessments of the level of anthropogenic forcing that should be avoided if rainforests are to be protected. International mitigation debates have often sought a global temperature threshold to which warming should be restricted, and 2°C has emerged as a benchmark for danger. Projections presented as a function of global mean temperature increase (*ΔT*_g_) are therefore particularly valuable from a political perspective, and could inform judgements of whether 2°C is a safe limit for African rainforests.

Existing analysis of climate change projections for Central Africa is very limited, and studies of African futures have sometimes omitted this region owing to the lack of observational data for model validation (e.g. http://www.knmi.nl/africa_scenarios/). Projections for the global tropics suggest that convective regions will become wetter in response to global warming [2] owing to an increase in atmospheric boundary layer moisture [3]; but this will be partly offset by a weakening of tropical circulation [4] and may be reversed locally by regional dynamics. The Intergovernmental Panel on Climate Change projections for Central Africa show small positive precipitation anomalies, based on data from the Coupled Model Intercomparison Project phase 3 (CMIP3) [5]. However, during the late twentieth century some western African forest regions experienced a drying trend [1], and some climate models project dryer futures at the Atlantic coast [6]. Hence, there is considerable uncertainty about the direction of precipitation change resulting from anthropogenic forcing, and this further creates uncertainty about the magnitude of local warming, which is likely to be modified by precipitation through cloud cover and/or soil moisture feedbacks.

Several studies have used the CMIP3 models to investigate the implications of specific *ΔT*_g_ thresholds for African precipitation [7] and the impact of these precipitation changes on the potential distribution of humid tropical forests [8]. These papers reveal that the increase in precipitation in Central Africa projected by many of the models is enhanced from 2°C to 3°C to 4°C of global warming, suggesting greater potential for rainforest expansion as global warming progresses. However, some models show drying in western regions, which could be associated with forest retreat, especially at 4°C. This is further complicated by projected increases in local temperature, which could allow rainforests to move into regions with higher elevations, but might also have an influence via changing rates of transpiration, photosynthesis and respiration.

The present study aims to explore trends in Central African temperature and precipitation associated with *ΔT*_g_, adding to previous work by comparing CMIP3 with two perturbed physics ensembles (PPEs) developed by the Met Office Hadley Centre (MOHC). Indices of specific relevance to tropical rainforests are used, and the range of model projections is presented for regions of interest, including west equatorial Africa where many models show a dry signal. Dynamics associated with contrasting precipitation responses in this region are examined as a first step to assessing the plausibility of a drying trend.

The model ensembles are described in §2, followed by an outline of the methodology. Section 3 presents the regional climate projections, and §4 assesses global fields from a subset of models in order to evaluate the mechanisms associated with drying. The implications of projected changes are then discussed.

## 2. Material and methods

### (a) Data

#### (i) Coupled Model Intercomparison Project phase 3

Monthly data were selected from CMIP3 [9] for all 19 models run in SRES A2 (see the electronic supplementary material, table S1), and the same models run in 20C3M, which incorporates historical forcings, available at: http://esg.llnl.gov:8080/index.jsp. These data were interpolated to a 1° × 1° spatial resolution.

#### (ii) Atmospheric Slab model Perturbed Physics Ensemble

Atmospheric Slab model PPE (AS-PPE) consists of 280 versions of HadSM3, which has the same atmospheric and land surface physics as HadCM3 (one of the coupled models included in CMIP3), but with a 50 m thermodynamic mixed layer or ‘slab’ ocean. Each model version was run in equilibrium experiments, with preindustrial and doubled CO_2_ (2× CO_2_). The runs continue for 20 years after equilibrium, and 20-year monthly means from this period were used for the analysis. These data have a spatial resolution of 2.5° latitude and 3.75° longitude, and 19 vertical levels.

Thirty-one atmospheric and land surface parameters were perturbed [10], with the intention of sampling uncertainties rather than producing a match to observations. Model versions with large top of atmosphere flux imbalances (greater than 5 Wm^−2^) and entrainment coefficients outside the likely range (less than 2 or greater than 4) [11] were excluded from the analysis, leaving 112 members. Data are available via the British Atmospheric Data Centre (BADC) at http://tiny.cc/qump_oxford (http://tiny.cc/badc_access).

#### (iii) Atmospheric Ocean model Perturbed Physics Ensemble

Atmospheric Ocean model PPE (AO-PPE) consists of 17 versions of HadCM3. The 17 parameter combinations were chosen from the possible 280 explored in AS-PPE and then coupled to a dynamical ocean. Each model version was run in transient experiments: a 150-year control run with preindustrial (1860) forcings and SRES A1FI. For the preindustrial, a 20-year period was selected 40 years into the run, to avoid spin up issues.

One of the 17 versions has the standard parametrizations of HadCM3, and this version is identical to that included in CMIP3 except for the addition of flux adjustments and an interactive sulfur cycle. Another version was chosen because it had the best simulation of present-day climate, and the other 15 were selected to span a range of climate sensitivities and parameter values [12]. Four of the model versions have entrainment coefficients greater than 4, which is outside the likely range, and these are excluded from the analysis, leaving 13 members. Data are available via the BADC as above.

### (b) Methods

Changes in annual temperature, annual precipitation and maximum climatological water deficit (MCWD_100_) were calculated, as these have been found to be good indicators of humid tropical forest extent [8]. Local changes were related to global mean temperature increase (*ΔT*_g_). Area-averages were used to explore the range of model projections for regions with contrasting signals in the ensemble means.

The different ensembles were used for different stages of this analysis. CMIP3 and AO-PPE were used to evaluate local change as a function of *ΔT*_g_, as these models were run in transient scenarios, with a change in anthropogenic forcing (and global temperature) over time. In AS-PPE, each model has a fixed level of anthropogenic forcing (2× CO_2_); therefore, it is not possible to directly investigate the impact of changes in global temperature that arise from different levels of anthropogenic forcing. However, this ensemble has a larger number of members and was used to explore inter-model variability.

#### (i) Maximum climatological water deficit

MCWD is calculated from monthly mean precipitation (*P*) and evapotranspiration (*ET*; mm month^−1^). The climatological water deficit (CWD) is computed for each month as follows. First, the month with the highest mean precipitation is identified and CWD for this month is set to 0, on the assumption that soil is saturated. For each month (*n*) that follows, CWD is computed from the difference between precipitation and evapotranspiration, and the previous month's CWD:
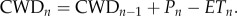
If CWD*_n_* > 0, then CWD*_n_* is set to 0. The MCWD is the most negative value of CWD
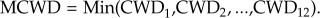


For multi-year periods, the calculation is applied to long-term monthly means. Here, *ET* is set at 100 mm month^−1^. Previous studies have used this approximation for calculating MCWD and shown skill in reproducing historical tropical vegetation distributions in Africa [8]. Changes in MCWD_100_ represent modifications in dry season water deficit owing to precipitation change alone (assuming constant evapotranspiration).

#### (ii) Rate of local change per °C of *ΔT*_g_

For CMIP3 and AO-PPE, changes in temperature, precipitation and MCWD_100_ for each model grid-point were linearly regressed against global mean temperature increase (*ΔT*_g_), using ordinary least squares and the slope of the regression line (*m*) used to represent local change in each index per °C of *ΔT*_g_. The regression slope (*m*) was calculated separately for each model and the ensemble mean. The data points were overlapping 20-year time periods: area-weighted global mean temperature anomalies and local anomalies of temperature, precipitation and MCWD_100_ associated with 2000–2019, 2010–2029, etc. (see the electronic supplementary material, table S2), relative to control climatologies (20-year preindustrial for AO-PPE, 1980–1999 for CMIP3). The slope was tested for difference from zero using a *t*-test, following Wilks [13], at a significance level of 5%. The percentage of models with positive slopes was calculated on a grid-point by grid-point basis to illustrate model agreement on the direction of change, and the percentage of models with significant slopes was computed to show agreement on a lack of signal.

#### (iii) Regional change associated with *ΔT*_g_

Area-weighted area averages of temperature, precipitation and MCWD_100_ anomalies were calculated for regions identified as exhibiting contrasting trends in the gridded ensemble mean projections: ‘west equatorial Africa’ (11–18° E; 10° S–5° N) and ‘central equatorial Africa’ (23–32° E; 10° S–5° N). For AS-PPE, the changes associated with 2× CO_2_ were used (based on 20-year means for the control and 2× CO_2_ scenarios). For CMIP3 and AO-PPE, changes associated with specific *ΔT*_g_ thresholds (1°C, 2°C, 3°C, etc.) were calculated.

In order to sample at each *ΔT*_g_ interval, annual *ΔT*_g_ time series were created for each model (see the electronic supplementary material, figure S1), using a 15-year control climatology (preindustrial for AO-PPE, 1985–1999 for CMIP3). These time series were smoothed using polynomial regression, and then the date at which the regression line crossed 1°C intervals of *ΔT*_g_ was extracted and defined as the median of a 15-year sampling period (see the electronic supplementary material, table S3). Anomalies of temperature, precipitation and MCWD_100_ were calculated by differencing these 15-year climatologies from control climatologies (preindustrial for AO-PPE, 1985–1999 for CMIP3). Note that the number of *ΔT*_g_ levels available varies between models and is a maximum of 6°C for AO-PPE and 3°C for CMIP3.

A statistical significance test of difference between the control and each *ΔT*_g_ interval was computed for each model to establish whether the response to warming is distinct from inter-annual variability. The Mann–Whitney *U*-test was chosen as precipitation is a skewed distribution in many arid regions and the test makes no assumptions about distribution. The direct method was applied due to the small sample size (15 years): for each year in the control, the number of years in the forced time period with a smaller value is counted, and the sum of these counts becomes the *U* statistic (*U*_1_). This is repeated for the forced time period to get *U*_2_, and the smaller of *U*_1_ and *U*_2_ is compared with the critical value. The significance level was set at 5%.

## 3. Projected trends in regional climate

**Figure 1.**
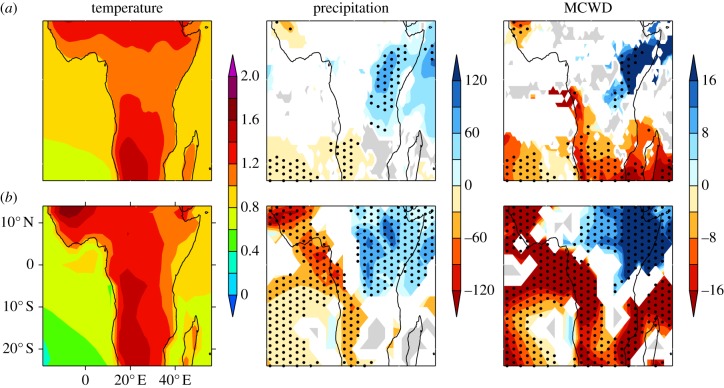
Maps of local ensemble mean change per °C of *ΔT*_g_: annual temperature (°C per °C), annual precipitation (mm yr^−1^ per °C) and MCWD_100_ (mm month^−1^ per °C) in CMIP3 (*a*) and AO-PPE (*b*). For temperature, locally insignificant (5% level) anomalies are masked in white. For precipitation and MCWD_100_, white indicates less than 66% model agreement on the direction of change, grey indicates more than 66% of models show no significant change and stippling indicates greater than 80% model agreement on the direction of change.

The African tropics are projected to warm more than the global average in all three ensembles (figure 1*a*; AS-PPE not shown). In CMIP3 the largest warming is in the subtropics, in contrast to AO-PPE, in which there is also pronounced heating (greater than 1.4°C per °C of *ΔT*_g_) in the south of Central Africa and in West Africa.

Precipitation changes demonstrate substantial variation between and within the ensembles (figure 1*b*). In general, AO-PPE projects larger changes per °C of *ΔT*_g_ than CMIP3. Both ensemble means show positive annual mean precipitation anomalies in East Africa, and negative anomalies in some western regions, with greater than 80% model agreement in the direction of change. The dry signal is much more extensive and of a larger magnitude in AO-PPE, at greater than 90 mm yr^−1^ °C^−1^ in parts of West Africa and west equatorial Africa in the ensemble mean. Both ensembles show agreement for a lack of signal over parts of Mozambique and Madagascar.

The spatial distribution of MCWD_100_ anomalies (figure 1*c*) is similar to that of annual precipitation (figure 1*b*), with most models projecting dry signals in western regions, and wet signals in the Horn of Africa. Relative to figure 1*b*, negative anomalies occupy a larger proportion of the African tropics, including the south and west of the Congo Basin, where there is a negative anomaly greater than 16 mm month^−1^ per °C of *ΔT*_g_ in the AO-PPE ensemble mean. This means there is inter-model agreement that dry seasons will intensify in regions where models agree that there will be no significant change averaged across the annual cycle (grey area in figure 1*b*), or disagree in the direction of mean change (white area in figure 1*b*). Therefore, even where there is considerable uncertainty about how the wet and dry season changes will average, there is consensus that precipitation will be reduced during dry seasons.

**Figure 2.**
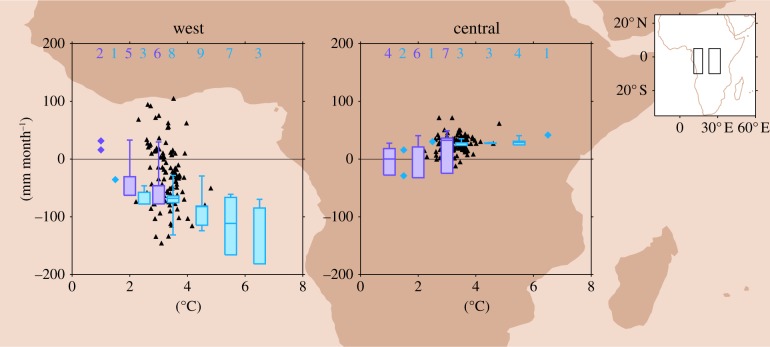
Regional MCWD_100_ anomalies (mm month^−1^) for ‘west equatorial Africa’ and ‘central equatorial Africa’; associated with 2× CO_2_ in AS-PPE (triangles), and *ΔT*_g_ levels for CMIP3 and AO-PPE (purple and blue boxplots, respectively: minimum, lower quartile, median, upper quartile and maximum). Only models with significant change (5% level) are included in the boxplots, the number of which is indicated. Where fewer than three models show significant change, boxplots are replaced with points for individual models. Boxplots based on all models are shown for comparison in the electronic supplementary material, figure S2. Note that not all models are available at each *ΔT*_g_ interval. Alternative plots based on the regression slope for all models are shown in the electronic supplementary material, figure S3.

The ensemble mean plots for both annual precipitation and MCWD_100_ suggest a contrast in the precipitation signal between western and eastern equatorial regions. Area-averages of ‘west equatorial Africa’ and ‘central equatorial Africa’ (figure 2) illustrate the spread of CMIP3 and AO-PPE model responses in each of these regions, and show how local changes develop as global temperature increases. Changes in AS-PPE associated with 2× CO_2_ are also shown. In central equatorial Africa, most models show little change or a modest wet signal. At 4°C and beyond, none of the models shows negative anomalies. In west equatorial Africa, there is a great deal of spread between members of AS-PPE in the direction and magnitude of change, although more models exhibit a dry signal than a wet signal. In the transient ensembles, there is little significant change at 1°C, but from 2°C there is a progressive decrease in MCWD_100_ and from 3°C this is greater than 100 mm month^−1^ in some versions of HadCM3. Note that the boxplots in figure 2 are based on a different number of models at each *ΔT*_g_ interval, but that individual models also show progressive change as global temperature rises.

The projections of annual precipitation and MCWD_100_ change presented here exhibit variation between models in terms of the direction, magnitude and spatial pattern of change; but there is one signal which emerges from many different models within the core rainforest zone: a drying in west equatorial Africa. There have been indications of such a signal in previous literature based on annual mean precipitation [6]. When measured here as MCWD_100_, the pattern shows consistency between models and the dry signal extends over a larger proportion of the Congo Basin. In §4, mechanisms associated with drying in this region are explored.

## 4. Dynamics associated with drying in west equatorial Africa

**Figure 3.**
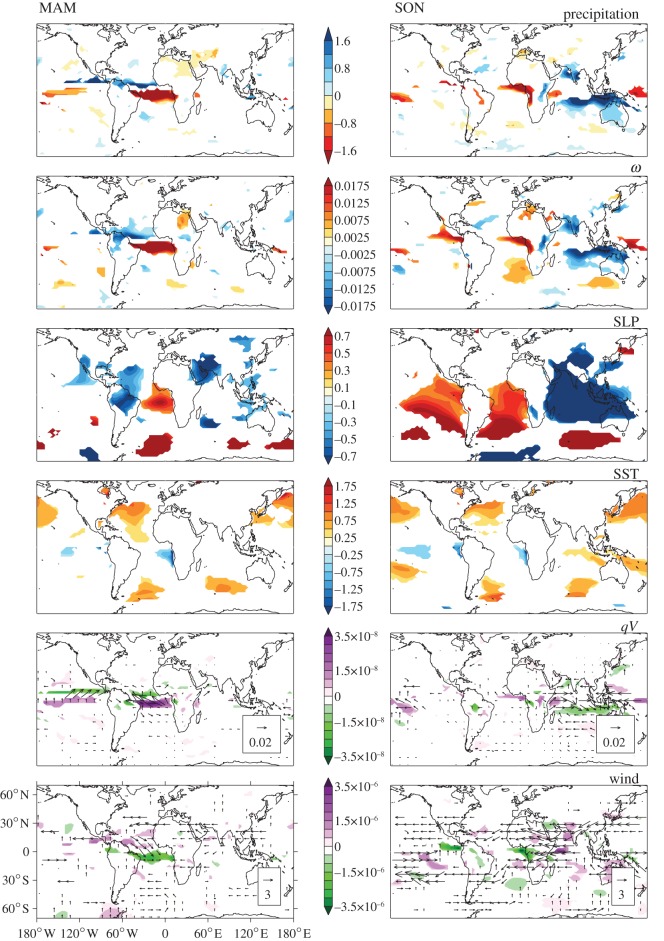
‘Dry’ minus ‘wet’ AS-PPE composite seasonal anomalies associated with 2× CO_2_ for precipitation (mm day^−1^), *ω* at 500 hPa (hPa s^−1^), SLP (hPa), SST (°C), *qV* at 925 hPa (kg kg^−1^ ms^−1^) with contours of moisture divergence (kg kg^−1^ s^−1^) and wind vectors at 200 hPa (m s^−1^) with contours of divergence (s^−1^), for MAM and SON. Areas not significantly different from zero (5% level) are masked in white.

The majority of models project an increase in dry season water deficit in west equatorial Africa, but AS-PPE also includes some models which show a wet signal in response to CO_2_ (figure 2). The slab model ensemble therefore provides the opportunity to investigate mechanisms associated with regional precipitation decline, through a comparison of members which show wetting and drying. The models with the 10 most negative and 10 most positive MCWD_100_ anomalies in west equatorial Africa were used to form a ‘dry’ and a ‘wet’ composite; and changes associated with 2× CO_2_ were explored for a range of atmospheric variables across the globe. Figure 3 displays the difference between the composites (dry–wet) in terms of precipitation, vertical velocity (*ω*), sea-level pressure (SLP), sea surface temperature (SST), wind, horizontal moisture flux (*qV*) and divergence.

These plots are based on averages of each group of 10 models, but grid-points are only shown where the difference between the dry and wet models is robust to variation within each composite according to the Mann–Whitney *U*-test. This test for consistency is important, as models with a similar MCWD_100_ response in ‘west equatorial Africa’ might have different regional precipitation signals and/or large-scale dynamical responses. The grid-points with significance in figure 3 represent signals which are shared by many models within the same composite, confirming that there are consistent differences between composites in Central African precipitation response.

Models in the dry composite exhibit negative precipitation anomalies in the west of the Congo Basin in the annual mean and all four seasons: DJF, MAM, JJA, SON (denoted by the initial letter of each month), albeit with limited spatial extent in MAM. The strongest dry signal occurs during SON. The same models have wet anomalies in East Africa in all seasons. By contrast, the wet composite shows positive precipitation anomalies throughout equatorial Africa, with the largest wetting at the Atlantic coast during SON. This regional precipitation difference in SON is clear from figure 3 and is matched by differences in *ω*, indicating that dry models experience a larger enhancement of subsidence over west equatorial Africa, and convection over East Africa.

There are also consistent distinctions between the dry and wet composites beyond the African tropics, suggesting that changes in large-scale dynamics may be at least partly responsible for the trends projected in this region. The tropical Atlantic and Indian Ocean could both be important.

### (a) Tropical Atlantic

The maps of difference in precipitation, *ω* at 500 hPa and *qV* at 925 hPa (figure 3) indicate that the precipitation reduction and downward anomalies in west equatorial Africa in the dry composite forms the eastern edge of a larger zonal band of subsiding air and moisture divergence at the surface which stretches across the Atlantic Ocean. To the north of this region of drying is a wet signal, suggesting a northward displacement of the Atlantic Inter-Tropical Convergence Zone (ITCZ). This is evident in all seasons (see the electronic supplementary material, figure S4) but has the largest anomalies and is most zonally oriented during MAM. It might be explained by a meridional contrast in SLP and SST anomalies, with more warming in the North than South Atlantic Ocean. Relative to wet models and the ensemble mean, dry models show cooling and pressure increases in the Gulf of Guinea, suggesting a more pronounced Atlantic cold tongue. This has previously been associated with changes in the latitude of the ITCZ and, as a consequence, precipitation changes in western Africa [14].

There may also be a connection with South America. Hadley Centre models are known to dry the Amazon in response to global warming [15], and in AS-PPE the ensemble mean, wet and dry composites all have negative precipitation change in this region; but the dry composite shows the largest reduction in precipitation. The anomalous subsidence which the dry models experience in the Atlantic Ocean throughout the year extends into the Amazon during DJF (see the electronic supplementary material, figure S4). This adds an additional motivation for diagnosing the mechanisms in these models, as the same processes might dry both rainforest regions. The Amazon dieback has been attributed to an El Niño-like state in HadCM3 [16], and drying in Central Africa on inter-annual timescales has also been linked to El Niño [17]; but there is no clear El Niño-like difference between dry and wet models.

### (b) Indian Ocean

There are also large differences between the composites in the Maritime Continent and eastern Indian Ocean, although these are only significant during SON (figure 3). Dry models show much more moistening associated with 2× CO_2_, and this is accompanied by a larger warming of SSTs, a convergence of moisture and decrease in pressure at the surface, and upward *ω* anomalies, suggesting an augmentation and westward extension of the warm pool region.

Links between the Indian Ocean and African precipitation have been found in previous studies, with particular emphasis on East Africa and the Sahel. Idealized Indian Ocean warming experiments, designed to diagnose teleconnections with the Sahel in JAS, have also shown a strong drying response in west equatorial Africa [18,19]. Hoerling *et al*. [19] attribute this to anomalous zonal circulation with rising air over the Indian Ocean and subsiding air over western Africa.

The dry models used here show evidence of a shift in zonal circulation during SON, in terms of *ω* anomalies, and horizontal fluxes in the Indian Ocean, with westerly flow at 925 hPa and easterly flow at 200 hPa, as well as convergence in the upper atmosphere over west equatorial Africa and the Gulf of Guinea (figure 3). The high SLP over the Atlantic Ocean could potentially be amplifying the strength of these anomalies, in keeping with Balas *et al*.'s [17] suggestion that an opposition between Indian and Atlantic Ocean SSTs might cause an eastward shift in convection, drying western Africa and wetting eastern Africa. Perhaps it is this contrast between ocean basins which is key: the difference between Maritime Continent (75–130° E; 10° S–10° N) and Gulf of Guinea (5° W–8° N; 10° S–3° N) SLP anomalies calculated for the 112 models in AS-PPE is positively correlated with MCWD_100_ anomalies in west equatorial Africa, during SON (*r* = 0.61) and the annual mean (*r* = 0.72). The correlation with Maritime Continent anomalies alone is weaker.

### (c) Discussion

The composite analysis therefore implies that drying in west equatorial Africa is associated with a zonal SLP anomaly between the Gulf of Guinea and the Maritime Continent and a shift in zonal circulation. However, further research is necessary: to establish whether there is a causal connection, to investigate reasons why the models might project differences between the Atlantic and Indian Oceans and to examine the plausibility of the response.

Part of the explanation for the signal may lie in the preindustrial climatology. There are contrasts between the dry and wet composites before any doubling of CO_2_, in both the Gulf of Guinea and the warm pool region (see the electronic supplementary material, figure S5). This is likely to influence the response to greenhouse gases. For example, the dry models have more precipitation in the warm pool region during the preindustrial, so it is not surprising that they show greater enhancement of convection under 2× CO_2_, as wet regions are expected to become wetter in a warmer climate [3]. Analysis of model biases in the preindustrial might therefore be a useful means for identifying implausible signals. In the case of the Maritime Continent, both the dry and wet composite have large biases relative to the observed climatology (GPCP), so it is not possible to suggest that either is more realistic on this basis.

Another way to investigate the mechanisms in the dry composite would be to establish whether the same zonal anomalies are associated with drying in west equatorial Africa in AO-PPE and CMIP3. The slab models do not have a dynamical ocean, so any change in SSTs and teleconnections are due to atmospheric forcing. It would be useful to examine whether there is evidence for a zonal shift in models with a fully coupled ocean–atmospheric system.

Additional mechanisms must also be investigated, as the zonal SLP contrast explains a maximum of 50% of the inter-model variability in the precipitation response (*r*^2^ = 0.5 in the annual mean). Other changes in large-scale atmospheric dynamics, such as the north–south contrast in Atlantic Ocean SSTs, may be important. However, previous work suggests that variation between modelled precipitation responses over tropical landmasses is associated more with uncertainties in the representation of direct local responses to greenhouse gases than with remote teleconnections to SSTs [20]. For example, drying in west equatorial Africa might be associated with the representation of the land–sea contrast, which would influence the strength of low-level westerlies during SON. Analysis using regional climate models might help to disentangle the role of local and remote influences.

Establishing whether spatially coherent drying of west equatorial Africa is a plausible or likely response is therefore beyond the scope of this paper. However, given that the dry signal is produced by many different models, and a potential mechanism for precipitation decline has been identified, the implications of such a change will now be examined.

## 5. Potential implications for African rainforests

African rainforests are already close to the hydrological limits of closed canopy forest [21], and their resilience to increases in water stress is uncertain, particularly given that drier conditions would also heighten the risk of fire [22]. Drier periods in the last two to three millennia led some forests to become more open, more fire-prone and less carbon-dense [23]. It is difficult to judge whether the magnitude of MCWD_100_ change projected here would drive this scale of ecological change. The average MCWD_100_ for humid tropical forests in Africa is approximately −300 mm month^−1^, and regions with MCWD_100_ below −400 mm month^−1^ are generally inhabited by other types of forest or savanna [8]. Therefore, the order of magnitude of the anomalies projected by some AO-PPE models after 2°C of global warming could have a large impact: at 3°C one model exhibits a decrease greater than 100 mm month^−1^, and at 4°C three models project change of this amplitude.

These changes are averaged over 15 years, and do not provide any information about the magnitude or frequency of single drought years, which have been shown to exert a damaging influence on rainforests in Amazonia during the last decade [24]. A tentative analysis of AO-PPE precipitation trends during JJA, the season with the most drying, suggests that there is little modification in inter-annual variability with warming: the change in standard deviation is less than 0.09 per °C of warming in all models. Thus, there is a shift in the mean with little to no change in variability, implying an increase in the intensity of dry years, which warrants further research.

Temperatures are also projected to increase, and in the AO-PPE ensemble mean this warming is greater than 1.4°C per °C of *ΔT*_g_ in the southwest of the Congo Basin (figure 1*a*). The vulnerability of rainforests to higher temperatures is debated. Warming may have a direct effect on rainforest trees by inhibiting photosynthesis, but the thermal tolerances of most species are poorly understood, as is the extent to which the influence of temperature will be overridden by CO_2_ enrichment [25]. Higher temperatures would also have hydrological implications, enhancing transpiration through higher leaf-to-air vapour pressure deficits. This could potentially amplify the projected increase in dry season water stress, which has been calculated here assuming fixed evapotranspiration. Although increasing CO_2_ concentrations are expected to increase plant water use efficiency, decreasing transpiration, initial assessments indicate that the net impact of higher temperature and higher CO_2_ will be an increase in evapotranspiration [15].

The projected dry signal therefore represents a potential risk to rainforests in the west of the Congo Basin, but it is unclear how resilient the forests would be to this precipitation forcing, especially given interactions with changing temperature and CO_2_. There is further uncertainty about how vegetation changes would feedback to regional climate, via modifications to surface roughness, albedo and evapotranspiration. If precipitation decreases were large enough to cause a reduction in tree cover, there might be less evapotranspiration and more run-off into rivers, leading to lower precipitation rates. However, the models used here do not incorporate dynamic vegetation, with the result that vegetation cover, and therefore evapotranspiration levels, are maintained even under very low precipitation levels.

The precipitation projections presented here might therefore differ if the models included dynamic vegetation and a more advanced land surface scheme, although it is not clear whether this would dampen or enhance the dry signal. Betts *et al*. [26] find that dynamic vegetation in HadCM3 amplifies drying in Amazonia, but not in the Congo Basin. The newest generation of global model experiments (CMIP5) includes several Earth System Models with dynamic vegetation, and existing research has largely found projections from CMIP5 to be similar to CMIP3 [27,28]. However, there has been little work to date to differentiate the influence of dynamic vegetation and this represents an opportunity for further research.

## 6. Conclusions

Many climate models project increases in precipitation in East Africa and decreases in western Africa in response to global warming. In west equatorial Africa, some models show large increases in dry season water deficit at 2°C (up to 78 mm month^−1^), with potentially dangerous implications for African rainforests. This is amplified at 4°C (up to 124 mm month^−1^) and beyond (up to 180 mm month^−1^) and is associated with pronounced local warming. In a subset of slab models, the dry signal in west equatorial Africa is found to be at the eastern edge of a large region of subsidence in the Atlantic Ocean. This may be associated with a northward shift of the Atlantic ITCZ, and/or a rearrangement of the zonal circulation during SON, with anomalous ascent over the Maritime Continent and Indian Ocean, and anomalous subsidence over western Africa and the Atlantic Ocean. In some seasons, the subsidence extends to South America, suggesting that this could have an influence on both the Congo and Amazon rainforests. Further research to assess the validity, plausibility and likelihood of these mechanisms is a priority. This should be accompanied by investigation into the potential interactions with other atmospheric and vegetation changes, which are highly uncertain, and could act to dampen or enhance the ecological risks implied by changes in precipitation.
